# Wildlife markets, COVID-19 and totalitarianism: A comment on Cawthorn et al.

**DOI:** 10.1007/s13280-021-01568-0

**Published:** 2021-06-15

**Authors:** Ronald I. Orenstein

**Affiliations:** 1825 Shady Creek Court, Mississauga, ON L5L3W2 Canada

Comment to: Cawthorn, D.M., A. Kennaugh, and S.M. Ferreira. 2021. The future of sustainability in the context of COVID-19. *Ambio* 50: 812–821. 10.1007/s13280-020-01430-9

Cawthorn et al. (2020) describe calls for the closure of wildlife markets as a global pandemic response (Orenstein 2020) as “totalitarian”. Although they claim to apply this term only in the context of Harari (2020), its pejorative implications may have the effect of delegitimizing the solution I (and others) have proposed. This comment is intended to clarify and contextualize our concerns.

There is now broad agreement among scientists that COVID-19 probably originated in a wildlife market in Wuhan, China, or along the chain supplying it with wild-caught or farmed wild animals. Infection of an intermediate species with a coronavirus from a bat, with subsequent mutation and recombination, is thought to have resulted in a form capable of infecting humans. A similar event was probably responsible for the outbreak of SARS in 2003.

Wild animals transferred to or sold in such markets are often kept in unsanitary and crowded conditions, under increased levels of stress, and likely have compromised immune responses. Urination and defecation from roosting or flying bats may have infected animals now held in the markets. Bringing together many species, some carried over long distances, increases the opportunities for cross-species infection.

Ever since SARS, virologists and epidemiologists inside and outside of China have been warning that allowing wildlife markets to operate increases the risk of further pandemics. One 2007 paper referred to such markets as a “time bomb” (cited in Orenstein, op. cit.). COVID-19 may have emerged because these warnings were not heeded. Failure to heed them now, even for well-intentioned policies including food security and poverty alleviation, could lead to another similar pandemic.

That is the justification behind calls for these markets, and the trade supplying them, to be permanently closed (Yang et al. 2020), a step being partially taken by the Government of China. This is not intended to stop subsistence consumption of wild meat. Rather, it is directed at large urban mixed-species wildlife markets, selling primarily mammals and birds and catering to an elite willing to pay high prices (as much as 30 times the price of chicken; Orenstein, op. cit.) for a luxury product.

The authors recommend, instead, an adaptive management approach. The problem with this recommendation—however valid in the long term—is that the necessary regulations and protocols do not yet exist, their drafting will take time, and their implementation is likely to be difficult. Viruses that could cause a new pandemic may not wait, and WHO, OIE and UNEP have recently called on authorities “to suspend the trade in live caught wild animals of mammalian species for food or breeding and close sections of food markets selling live caught wild animals of mammalian species as an emergency measure” (WHO, OiE, and UNEP, 2021) unless such effective regulations are in place.

The authors state that closing wildlife markets will not halt the spread of COVID-19. This is obvious, but closing the animal markets is a policy designed to reduce the chance of a similar outbreak in future. Objecting to closing wildlife markets immediately, given the history of SARS and COVID-19, is equivalent to objecting to turning a fire hose on a burning building on the grounds that what is really needed are updated fire control regulations and improved safety equipment.

The authors argue that closing markets will severely impact the livelihoods of the poor. However, the impact of another pandemic on the poor is likely to be far greater. The legal global wildlife trade, excluding seafood, furniture and fashion, is worth about $11 billion per year (Andersson et al. 2021). Rural communities may receive less than 1% of that. By contrast, the global cost of COVID-19 may be as much as $28 trillion over 5 years (Georgieva 2020). The impact on rural communities dependent on ecotourism has already been catastrophic (Rondeau et al. 2020; Spenceley et al. 2021).

The authors accuse advocates of a “myopic focus” on the wildlife trade as opposed to a broader range of anthropogenic and environmental drivers of zoonotic outbreaks. Recommending a specific urgent action to address the likeliest proximate cause of at least two pandemics, however, by no means rejects broader holistic solutions. A global approach is surely necessary (Roe and Lee 2021), but will take time to assess and implement. Time may not be on our side.

The authors contrast totalitarian governance with citizen empowerment, but overlook the possibility that closure of wildlife markets—which may threaten the health of far more people then they serve as clients—may actually reflect citizen empowerment. A substantial majority of respondents in Wuhan and Shanghai, for example, support permanent closure of wildlife markets (Orenstein, op. cit.).

A cooperative global effort is needed both to prevent, as far as is possible, another pandemic and to mitigate any consequences of preventative actions on the livelihoods, and indeed the lives, of the poor and those directly affected by these actions. Such issues, and their potential effect on people, deserve serious debate.
